# Low-intensity sciatic nerve-stretch injury increases nociception, anxiety-like behaviors, and astrocyte activity in male Wistar rats

**DOI:** 10.1590/1414-431X2025e14481

**Published:** 2025-05-30

**Authors:** G.K. Silva-Cardoso, P.E. Bello-Junior, W. Lazarini-Lopes, A.A. Ferrarese-Tiballi, C.R.A. Leite-Panissi

**Affiliations:** 1 Laboratório de Neurofisiologia da Dor e do Comportamento, Departamento de Psicologia, Faculdade de Filosofia, Ciências e Letras de Ribeirão Preto, Universidade de São Paulo, Ribeirão Preto, SP, Brasil; 2 Laboratory Neurophysiology, Department of Physiology and Biophysics, College of Medicine, Howard University, Washington, DC, USA; 3 Department of Pharmacology & Physiology, Georgetown University, Washington, DC, USA

**Keywords:** Chronic pain, GFAP, Nociceptive tests, Sciatic nerve-stretch injury (NSI)

## Abstract

The incidence of chronic pain in the general population is highly correlated to anxiety disorders, which promote negative effects on the quality of life. Stretch injury is the primary cause of nerve dysfunction and injury in the civilian population. Here, we characterized changes in nociception, anxiogenic-like behaviors, and astrocyte expression in the low-intensity sciatic nerve-stretch injury (NSI) model. Male Wistar rats were submitted to NSI, chronic constriction injury of the sciatic nerve (CCI), or sham surgery (SHAM). Animals were submitted to nociceptive tests (von Frey, acetone, and hot plate) before surgery and 6, 12, 18, and 24 days post-surgery. Anxiety-like behaviors were assessed in the open field test (OFT) 23 days after surgery. Immunofluorescence for astrocyte activity (glial fibrillary acidic protein (GFAP)) was performed in cortical, thalamic, and brainstem areas involved with pain and emotional processing. Animals submitted to NSI showed increased mechanical allodynia and thermal hyperalgesia, similar to those submitted to CCI. In the OFT, both NSI and CCI animals showed an increase in anxiety-like behaviors. Also, NSI animals presented an increased expression of GFAP in all analyzed areas, similar to CCI animals. In conclusion, the NSI model produced behavioral alterations comparable to those observed in the CCI model, including hypersensitivity to mechanical and thermal (heat) stimuli that lasted for more than three weeks. Additionally, both models induced a similar increase in GFAP expression in cortical, thalamic, and brainstem regions.

## Introduction

Chronic pain is a global problem that has reached epidemic proportions: 20% of adults have pain and another 10% are diagnosed with chronic pain every year (1,2). Diseases and injuries to peripheral nerves can trigger sensory and motor deficits and neuropathic pain that can cause lifelong disabilities, including emotional alterations such as anxiety (3). Although peripheral nerves undergo tensile loading under normal physiological conditions, abnormal conditions might induce peripheral nerve injury that can even affect the central nervous system (4).

Low-intensity lesions associated with nerve stretching such as ankle sprains affect 75-85% of the population (5), and tibial nerve injury occurs in around 80% of cases (6). Studies demonstrate that most patients can fully recover in the months following the injury, but 40% keep displaying pain symptoms (7). If lesions are not treated adequately, they may trigger a cascade of inflammatory responses (8), which might lead to chronic pain (9).

Notably, persistent pain conditions are often accompanied by emotional and cognitive disturbances (10). These dysfunctional or maladaptive changes in aversive/motivational circuitry likely contribute to pain management challenges (11). Furthermore, an increase in pro-inflammatory markers, such as astrocyte activity, is commonly detected in brain areas responsible for or associated with pain and emotional processing, indicating that neuroinflammation and pain are closely related (12). However, there is a lack of studies evaluating the long-term nociceptive, behavioral, and astrocyte changes that result from low-intensity sciatic nerve-stretch injury (NSI).

There are several animal models of neuropathic pain. Among them, surgical models, like the chronic constriction injury (CCI) of the sciatic nerve, are of fundamental importance in the induction of painful states (13). However, it is also essential to characterize changes in nociceptive and anxiogenic behaviors in low-intensity models of chronic neuropathic pain due to their high prevalence in the population (6). Therefore, we aimed to investigate whether the NSI model can promote changes in the sensory and emotional aspects of pain similar to those observed in animals submitted to the CCI model. Furthermore, we aimed to investigate changes in astrocyte activity by measuring glial fibrillary acidic protein (GFAP) expression in brain regions associated with pain perception and emotional behaviors, including the ventral posteromedial nucleus of the thalamus (VPM), primary sensory cortex (S1), and ventrolateral periaqueductal gray matter (vlPAG).

## Material and Methods

### Animals

Experiments were performed using 24 male Wistar rats (±250 g; eight weeks old at the beginning of the experiments) obtained from the animal facility of the Faculty of Medicine of Ribeirão Preto, University of São Paulo (Brazil). Experimental groups were SHAM (n=8), CCI (n=8), and NSI (n=8). All animals were kept in home cages (4 rats/cage) of 35×19×25 cm polypropylene lined with shavings in controlled temperature (24±1°C) and with water and food *ad libitum*. Experimental protocols were carried out in compliance with the recommendations of the Conselho Nacional de Controle de Experimentação Animal - Ministério da Ciência e Tecnologia, Brazil, and received the approval of the Ethics Committee for Animal Use of the University of São Paulo at Ribeirão Preto campus, protocol number 2018.1.103.58.5.

### Experimental design

**Figure 1:**
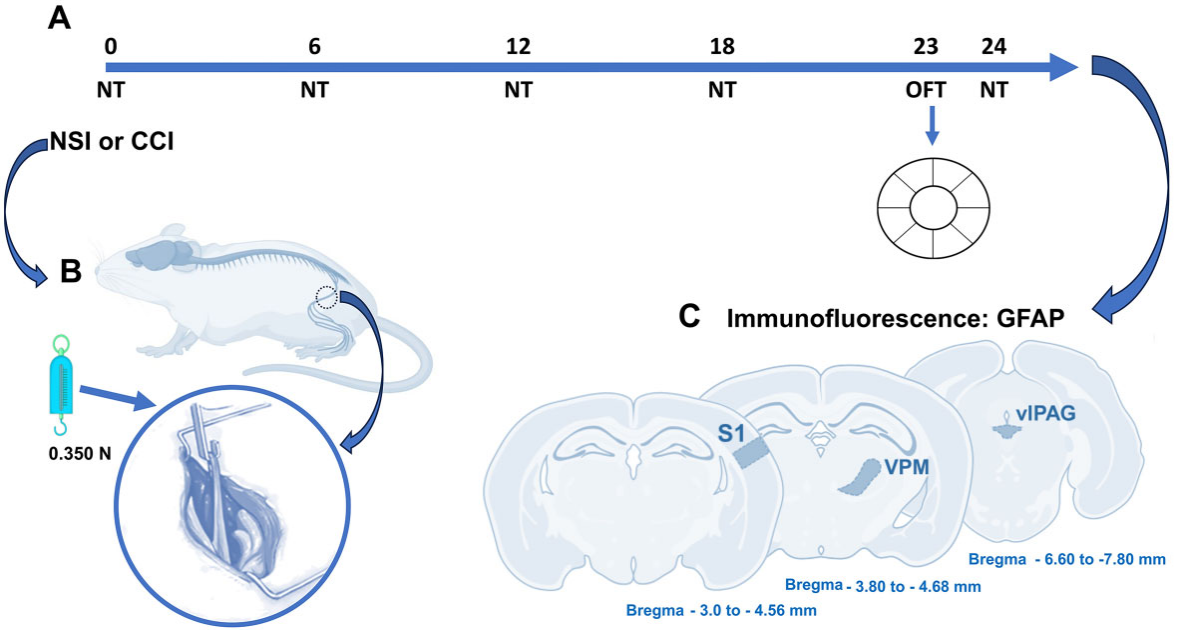
Experimental design. Animals were submitted to nociception testing (NT) (acetone, von Frey, and hot plate) at baseline. Then, animals underwent SHAM, CCI, or NSI surgery, and NT was repeated after surgery at 6, 12, 18, and 24 days. The OFT was performed on day 23 (**A**). Schematic representation of the sciatic nerve-stretch injury (NSI) (**B**). On day 24, tissue was collected for immunohistochemistry measurement of glial fibrillary acidic protein (GFAP) in brain regions (**C**). OFT: open field test; CCI: chronic constriction injury of the sciatic nerve model; SHAM: false operation control; VPM: ventral posteromedial nucleus of thalamus; S1: primary sensory cortex; vlPAG: ventrolateral periaqueductal gray matter.

Three researcher (G.K.S.-C., P.E.B.-J., and W.L.-L.) performed the behavioral experiments blinded to the test condition (using colors and numbers on the animals' tails), and testing groups were randomized. The von Frey, acetone, and hot plate tests were carried out between 9 am and 1 pm, with 10-min habituation and 10-min intervals between tests. The test sessions were performed before surgery (day zero - baseline) and 6, 12, 18, and 24 days after the surgery. The open field test (OFT) was carried out between 11 am and 1 pm, with 60-min room habituation before the test. Light intensity was measured in the center of the apparatus (60 lux), and the tests were recorded and stored for behavioral analysis. The apparatus was cleaned with 20% ethanol before all test sessions. See the experimental design in Figure 1.

### Sciatic nerve-stretch injury (NSI) model

Peripheral neuropathy was induced by the sciatic nerve stretch injury model (14). In summary, the rats were anesthetized with the association of 10% ketamine hydrochloride (75 mg/kg, Cetamin, Syntec, Brazil) and 2% xylazine hydrochloride (10 mg/kg, Xilazin, Syntec) administered intramuscularly. Afterward, animals were placed on a surgical table (dorsal position), a trichotomy was performed on the hind limbs, and the area was disinfected with iodo-polvidine (Rioquímica, Brazil). A 1.5-cm incision was made on the inner thigh, reaching the muscular fascia to separate the gluteal muscles and the biceps femoris muscle with exposure and rapid stretching of the sciatic nerve with a glass rod. The dissection was performed at the femur's small trochanter's height at length, measuring about 8 mm from the proximal sciatic nerve. The nerve lesion was performed by stretching the sciatic nerve with a sliding action of 2 cm, and the nerve was kept stretched for 3 min (0.350 N, using a spring-scale device). The muscles were then released and the skin was sutured with cotton thread. The sciatic nerve of the false-operation control group (SHAM) was not exposed. Finally, the epithelial tissue was sutured with 2.0 silk threads. The surgeries were performed between 9 am-3 pm, 70% alcohol was used to clean the table, and GermeRio (Rioquímica) was used to disinfect the surgical instruments before each surgery.

### Chronic constriction injury of the sciatic nerve model (CCI)

Peripheral neuropathy was induced by CCI of the sciatic nerve model as previously described (15,16). In summary, the rats were anesthetized, placed on a surgical table, and an incision of 1.5 cm was made on the thigh's inner side, with sciatic nerve exposure. The sciatic nerve was transfixed in 3/4 of its diameter, constricted in one place, and returned to its original position. In the end, the muscles were released, and the skin sutured with cotton thread. In the false-operated control group (SHAM), animals were submitted to the same general surgery procedures, but the sciatic nerve was not exposed.

### Mechanical and thermal sensitivity assessment

The mechanical sensitivity was assessed by an electronic von Frey device (Insight Instruments, Brazil). Increasing forces from the electronic filament were applied to the hind paw plantar surface until the paw was withdrawn (17). The mechanical paw threshold (in grams) was calculated as the mean of three values obtained in each session (17). The mechanical withdrawal threshold was assessed in the contralateral and ipsilateral paw to the surgery.

For the plantar acetone instillation test, which measures cold allodynia, animals were placed in acrylic boxes and 100 μL of acetone was instilled in the animal's paw with a small syringe. The behavior was measured for 40 s using the following score: 0 (no stimulus response), 1 (quick withdrawal or paw movement), 2 (repeated paw movement), and 3 (repeated movements of the hind paw and licking the paw) (17). The cold allodynia (in score) was calculated as the sum of three values obtained in each session (18).

The hot plate test assesses the latency for animals' reaction to a heated metal surface (50±1°C). Animals were placed on the heated plate, and the latency to thermal behavioral response (jump, withdrawal, or licking of the hind or front legs) was recorded. A cut-off time of 30 s was adopted to avoid possible injuries from prolonged heat exposure (17). The latency threshold (in seconds) was calculated as the mean value from three test sessions (18).

### Assessment of locomotor activity and anxiogenic-like behaviors

The OFT was used to evaluate the locomotion pattern and anxiety-like behavior on the 23rd experimental day. The OFT (a circle of 60 cm in diameter and 50 cm in height) was divided into internal and external quadrants. Animals were placed in the center of the apparatus and exploratory behavior was recorded for 5 min for behavioral analysis (18,19). We measured the time the animals spent in the center of the apparatus, the number of crossings between quadrants, the time of grooming, the time of freezing, and the time of elevation (19).

### Histological processing

On the 24th day, the animals were deeply anesthetized with 4% xylazine (30 mg/kg) and 10% ketamine (225 mg/kg) and perfused with 0.01 M phosphate-buffered-saline (PBS), followed by Somogyi's solution (8% paraformaldehyde in 0.1M phosphate-buffered + 4 mL of 25% glutaraldehyde + 300 mL of picric acid). The brains were removed and fixed in Somogyi's solution for 4 h, followed by tissue cryoprotection in a 30% sucrose solution. After that, the brains were frozen in isopentane and stored at −80°C in an anti-freezing solution (50% PBS, 30% ethylene glycol, 20% glycerol). The coronal sections for immunohistochemical analysis were cut in series of 40-μm thickness and were obtained from S1 (coordinates: −3.0 to −4.56 mm posterior to bregma), VPM (coordinates: −3.80 to −4.68 mm posterior to bregma), and vlPAG (coordinates: −6.60 to −7.80 mm posterior to bregma) (20).

### Immunofluorescence for GFAP and imaging

GFAP immunoreaction was performed as previously described (18,19). Briefly, tissues were washed in PBS and pre-incubated for 1 h in 10% normal goat serum (NDS, Jackson Immunoresearch Laboratories, USA) diluted in PBS + 0.3% Triton X-100. After that, the sections were incubated for 24 h in a mixture of primary antibody anti-GFAP (1:1000, Abcam (USA) ab190288, lot. GR3359366-7) at 4°C. On the second day, the tissue was washed in PBS, incubated for 2 h in a donkey anti-mouse IGg Rhodamine B (1:500, MiliporeSigma (USA) AP192R, lot. 2189681) and washed in PBS. The negative control sections were incubated without the primary antibody. Slides were mounted in Vectashield medium (Vector Laboratories, USA) and stored in a refrigerator.

Images were captured in an Olympus BX61VS microscope (Japan) and analyzed in 400× magnification. Six slices were analyzed per animal for each structure (n=6 animals randomly selected per group). The intensity was analyzed by the integrated optical density (IOD) method using the ImageJ software (https://imagej.nih.gov/ij/), as previously described (18). The mean value of the IOD (the product of area and average gray value) was calculated using the average value of 3 regions of interest (ROI) randomly established within each structure of each animal. The ROI area for the vlPAG was 2,500 μm^2^, while for the VPM and S1, the ROI area was 10,000 μm^2^. With this approach, the three ROI for each structure of interest nearly encompassed the entire structure.

### Statistical analysis

The Shapiro-Wilk test was used to test data normality. Parametric data from the nociceptive tests (von Frey, acetone, and hot plate) were analyzed by two-way ANOVA followed by Tukey's test for multiple comparisons. Parametric data from the OFT and immunofluorescence for GFAP expression were analyzed by one-way ANOVA, followed by Tukey's test for multiple comparisons. Parametric data are reported as means±standard error of the mean (SEM). The significance level was set at P<0.05 for all analyses. Figures and statistics were prepared using Prism software (version 8.0, GraphPad Software, USA).

## Results

### Comparative study between the NSI model and the CCI model

**Figure 2:**
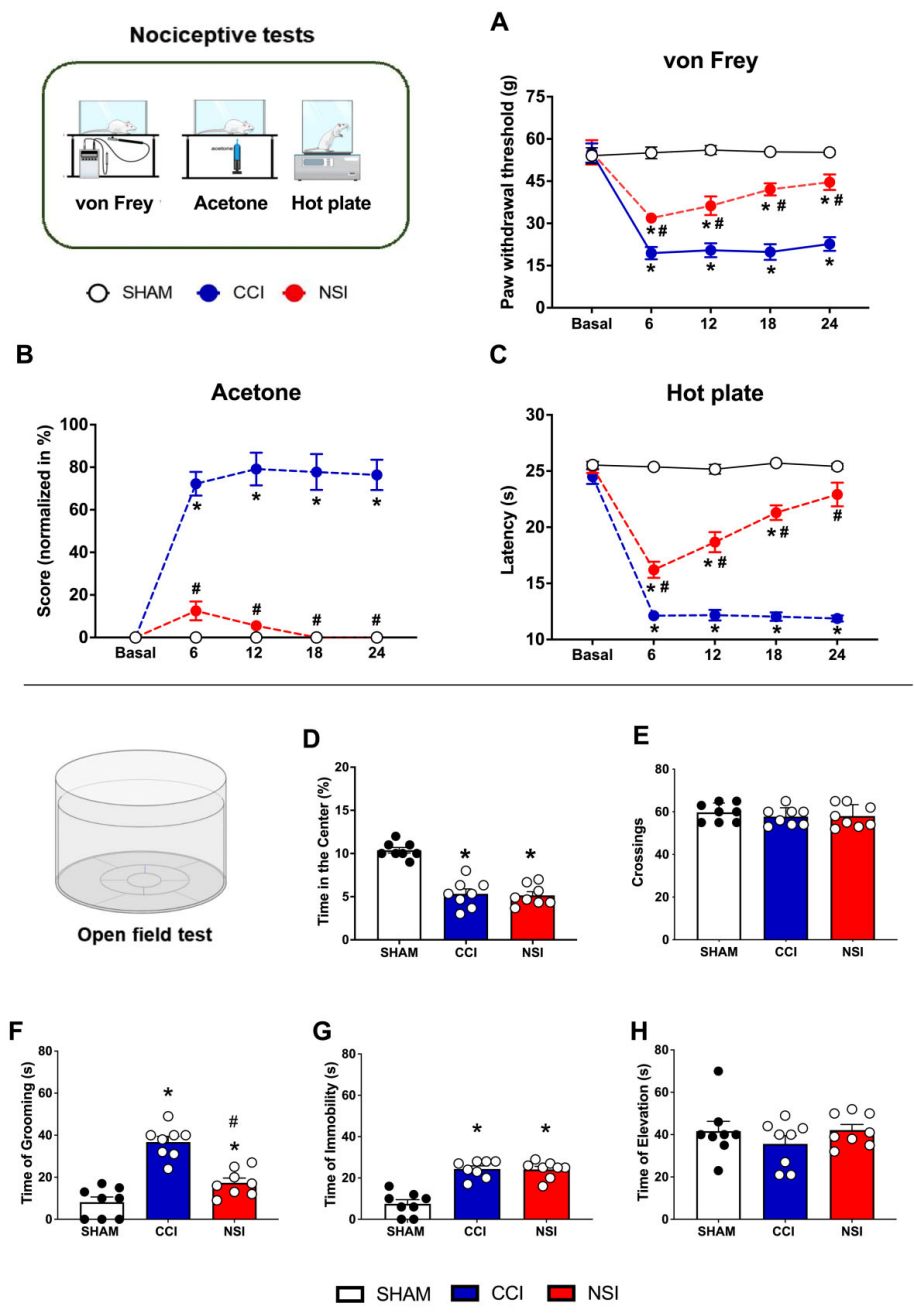
Assessment of mechanical withdrawal threshold, thermal allodynia, and hyperalgesia induced by sciatic nerve-stretch injury model (NSI). Results of mechanical sensitivity assessed by the von Frey test (**A**), and thermal sensitivity assessed by the acetone (**B**) and hot plate (**C**) tests of rats submitted to sham surgery (SHAM), chronic constriction injury (CCI), or nerve-stretch injury (NSI) at 6, 12, 18, and 24 days. **D-H**, Results of the open field test (OFT). **D**, Percent (%) time spent in the center of the open field. **E**, Total number of crossings. **F**, Time spent grooming. **G**, Time of immobility. **H**, Time spent rearing (elevation). Data are reported as mean±SEM; n=8/group. *P<0.05 SHAM *vs* NSI and CCI groups. ^#^P<0.05 NSI *vs* CCI group. Data from the nociceptive tests were analyzed using two-way ANOVA and data from the OFT were analyzed by one-way ANOVA followed by Tukey's post-test.

First, we performed a comparative study between the NSI model and the CCI model to observe possible nociceptive changes in a battery of nociceptive tests, including mechanical allodynia (von Frey), cold allodynia (acetone test), and hot plate (hyperalgesia). Time-dependent behavioral changes were examined in injured and uninjured rats by measuring mechanical and thermal thresholds on days 6, 12, 18, and 24 after NSI, SHAM, or CCI surgery (Figure 2A). The statistical analysis of the mechanical threshold obtained in the von Frey test for the NSI, SHAM, and CCI groups showed significance for the condition factor (F_2, 21_=1673, P<0.0001), time (F_4, 84_=203.1, P<0.0001), and the interaction between condition and time (F_8, 84_=82.83, P<0.0001). Tukey's post-test showed a significant difference (P<0.05) in the NSI and CCI groups compared to SHAM on days 6, 12, 18, and 24 (Figure 2A).

In the acetone test, a significance was found for the surgery factor (F_2, 21_=426, P<0.0001), time (F_4, 84_=27.33, P<0.0001), and interaction between surgery and time (F_8, 84_=23.63, P<0.0001). The Tukey post-test showed a significant difference (P<0.05) in the NSI group compared to the CCI group on days 6, 12, 18, and 24 (Figure 2B). No significant differences were observed between NSI and SHAM groups in the acetone test.

In the hot plate test, statistical analysis indicated a significance for the in the paw withdrawal threshold considering the time factor (F_4, 84_=91.27, P<0.0001), surgery (F_2, 21_=331.4, P<0.0001), and in the interaction between time and surgery (F_8, 84_=37.25, P<0.0001). Tukey's post-test showed a significant difference on thermal threshold (P<0.05) in the NSI group compared to the CCI group on days 6, 12, 18, and 24 (Figure 2C). The NSI group showed significant differences from the SHAM group on days 6, 12, and 18 (P<0.05); however, the measurement on day 24 was not significantly different from the SHAM group on the hot plate test.

### Open field test

A significant difference was found between the groups in the % time spent in the center of the apparatus (F_2, 21_=44.12, P<0.0001). The SHAM group was different compared to the NSI (P<0.001) and CCI (P<0.001) groups (Figure 2D). As for the number of crossings, no significant differences were observed between groups (P=0.65, Figure 2E).

In the evaluation of complementary measures of the OFT, significant differences were found in grooming time (F_2, 21_=34.66, P<0.0001, Figure 2F) and immobility time (F_2, 21_=15.33, P<0.0001, Figure 2G). The Tukey's post-test showed that the NSI and CCI groups were significantly different from the SHAM group (P<0.05). In addition, the grooming time was significantly different between the CCI and NSI groups. Finally, no significant difference was found in the elevation time between groups (P=0.42, Figure 2H).

### Immunoreactivity assessment of GFAP

**Figure 3:**
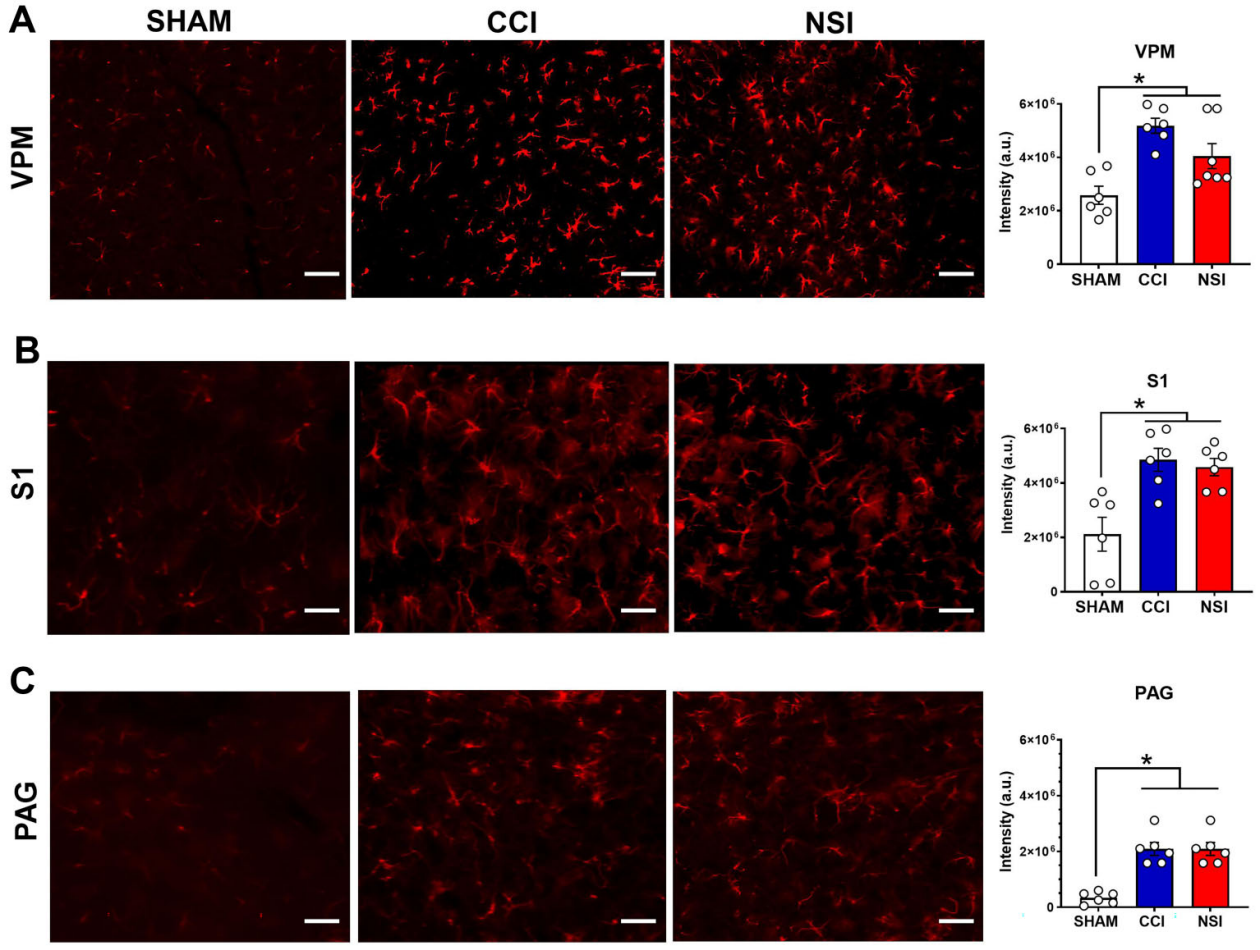
Immunofluorescence for glial fibrillary acidic protein (GFAP). Representative GFAP expression (red) in the VPM (**A**), S1 (**B**), and ventrolateral PAG (**C**) and corresponding graphs. Scale bar 50 μm. Data are reported as mean±SEM; n=6/group. *P<0.05 SHAM *vs* CCI and NSI; one-way ANOVA. a.u., arbitrary unit; CCI: chronic constriction of the sciatic nerve model; NSI: sciatic nerve-stretch injury model; SHAM: false-operation control; VPM: ventral posteromedial nucleus of thalamus; S1: primary sensory cortex; PAG: periaqueductal gray matter.

The immunofluorescence for GFAP in the VPM showed a significant value of the integrated density (F_2, 16_=10.85, P=0.001). Tukey's post-test revealed that the NSI and CCI groups were significantly different compared to the SHAM group (control, P<0.05, Figure 3A). Regarding the S1, a significant difference was found (F_2, 15_=10.17, P=0.001). The Tukey's post-test demonstrated that the SHAM group differed from the CCI and NSI groups (P<0.05, Figure 3B).

Finally, in the vlPAG region, a significant difference was found (F_2, 15_=26,79, P<0.0001); Tukey's post-test showed that the NSI and CCI groups were significantly different than the SHAM group (control, P<0.05, Figure 3C). We did not observe any difference between the NSI and CCI groups.

## Discussion

The results from the NSI group showed a reduction in the paw withdrawal threshold in the mechanical allodynia test (von Frey test), a threshold similar to the animals from the CCI model group, and this reduction persisted until the last time point of evaluation (24 days after surgery). In the hyperalgesia test (hot plate), latency to response decreased in the NSI animals in the first three evaluations (18 days after surgery). However, compared to SHAM animals, this reduction in the thermal threshold did not persist until day 24. Finally, in the cold allodynia (acetone test), we did not observe an increase in the response scores of NSI animals at any time point of evaluation. Therefore, the NSI induced mechanical and thermal (heat) allodynia, but unlike the CCI model, the NSI did not induce cold allodynia in rats.

The literature demonstrates that peripheral nerve injury by chronic constriction of the sciatic nerve (CCI model) generates anxiety-like behaviors in animals (18,21). NSI animals spent less time exploring the open field center and more time with grooming and immobility. The NSI group presented an anxious behavior similar to the animals from the CCI group. Anxiety is a psychopathology frequently observed in patients with neuropathic pain, which complicates treatment and severely impairs quality of life (22). Therefore, a model of chronic pain that mimics this characteristic has the advantage of allowing for assessment of emotional comorbidities associated with chronic pain.

Several studies have demonstrated the involvement of glial cells in pain induced by peripheral injuries (12,23). Microglia and astrocytes are essential for initiating and maintaining pathological pain (24). We observed in our study that animals submitted to the NSI model showed an increase in astrocyte expression (GFAP) in a cortical region, the S1, in a thalamic nucleus, the VPM, and in the vlPAG, a brain area located in the lower brainstem.

Studies in animals and humans suggest that maladaptive changes in S1 are commonly associated with chronic pain (25). It has recently been reported that structural and functional plasticity in cortical regions associated with nociception contributes to chronic pain states (26). These neuroplastic alterations include increased excitability or remodeling of synapses in the primary (S1) and secondary (S2) somatosensory cortex (25,27). A similar study showed that astrocyte-mediated growth of dendritic spines in S1 accompanies the initial transition phase to persistent allodynia after sciatic nerve injury (28). In line with these data, we observed an increase in GFAP expression in S1, supporting the role of astrocyte signaling into the S1 in the manifestation of chronic pain.

The VPM receives projections from the medullary dorsal horn and is mainly involved in the sensory-discriminative aspects of pain (29). Alterations of astrocytes in the thalamus of animals with injured peripheral nerves have already been demonstrated (30). Therefore, our data about GFAP in the VPM of animals submitted to the NSI are in line with previous studies in other models of chronic pain. These results align with the literature on sciatic nerve injuries that induce not only peripheral changes but also cortical and subcortical changes in the central nervous system (31).

The PAG is a critical regulatory center for supraspinal pain and, together with the thalamic nuclei, is fundamentally involved in the downward modulation of noxious mechanical and heat-induced responses (32). Preclinical studies of nerve injury models have demonstrated glial activation in the PAG (33). The vlPAG, through the spinal projection of the sciatic nerve (34), has a significant role in the descending control of noxious via afferent connections with the rostral ventromedial medulla (35). Our results also revealed an increase in astroglia activation in vlPAG columns of animals submitted to the NSI model, similar to levels observed in the CCI model. Additionally, unilateral sciatic nerve injury (NSI or CCI model) induced bilateral activation of astrocytes in the vlPAG. These results are likely related to bilateral spinal cord projections for vlPAG responses, which appear to be predominantly bilateral and non-somatotopic (36).

The CCI experimental model in rats induces pain-related behaviors similar to those observed in humans, being accepted as a model that resembles human neuropathic pain (15). This was the reason why we adopted this model as a positive control for nociceptive and behavioral measurements. This model reduces almost all Aβ and most Aδ fibers, which are axotomized, while large numbers of C fibers remain intact (37). However, the CCI is invasive and demands greater manipulation of the peripheral nerve, which results in the loss of nerve fibers. The NSI model, characterized by a low-intensity sciatic nerve stretch, showed the same phenotype as the neuropathic pain models without constricting or cutting the nerve.

One limitation of the present study is that we did not perform histological analysis of the sciatic nerve. However, a similar study showed that the nerve traction injury model in the rat sciatic nerve impairs not only the motor and sensory functions of the nerve, but also the axonal integrity (14). The authors demonstrated that the traction force (0.7 N) promoted damage by a 20% elongation of the sciatic nerve, and after two weeks, functional recovery was approximately 85% (14). Since we used a traction power of 0.350 N, we can infer that the injuries induced in our study were less intense, generating less stretching of the sciatic nerve and, consequently, a lower injury rate than in the study of Byun and Ahn (14). A second limitation of our study was that we did not use females. Robust evidence indicates that males and females have different mechanisms for acute and chronic pain processing (38). Therefore, future studies focusing on female rats and different phases of the estrous cycle will undoubtedly expand the knowledge about how low-intensity injuries (NSI model) can modulate the aspect of sensory discriminative and emotional chronic pain.

Animal models of neuropathic pain are fundamental for developing effective treatments. Therefore, animal models in pain research must mimic the human condition, with careful consideration of the model's predictive validity for the human clinical situation being investigated. Also, in chronic pain, structural and functional brain changes and negative affective states are associated with alterations in multiple brain areas, such as the corticolimbic circuit (39). Reactive astrocytes in cortical regions related to emotion regulation are involved in chronic pain and induce emotional alterations (29). Therefore, our study reinforces that low-intensity injuries can generate pain chronification similar to high-intensity injury models, such as the CCI. Additionally, although NSI animals presented a faster recovery of their nociceptive thresholds, they displayed the same levels of anxiety and astrocyte expression as the CCI group.

We demonstrated that animals submitted to the NSI model presented the same behavioral alterations of the gold standard model used to assess chronic pain, the CCI. These alterations included mechanical and thermal (heat) hyperalgesia and increased anxiogenic-like behavior. Finally, a similar increase in GFAP expression was observed in cortical, thalamic, and subthalamic areas in both the NSI and CCI models, supporting the hypothesis that increased astrocytic signaling in these brain areas may underlie the reduced nociceptive thresholds as well as the increased anxiety-like behavior in models of chronic pain.
